# Somatotopic Organization of Hyperdirect Pathway Projections From the Primary Motor Cortex in the Human Brain

**DOI:** 10.3389/fneur.2022.791092

**Published:** 2022-04-25

**Authors:** Sonia Pujol, Ryan P. Cabeen, Jérôme Yelnik, Chantal François, Sara Fernandez Vidal, Carine Karachi, Eric Bardinet, G. Rees Cosgrove, Ron Kikinis

**Affiliations:** ^1^Surgical Planning Laboratory, Department of Radiology, Brigham and Women's Hospital, Harvard Medical School, Boston, MA, United States; ^2^Laboratory of Neuro Imaging, Stevens Institute for Neuroimaging and Informatics, Keck School of Medicine of the USC, University of Southern California, Los Angeles, CA, United States; ^3^Sorbonne Université, CNRS, INSERM, APHP GH Pitié-Salpêtriére, Paris Brain Institute - Institut du Cerveau (ICM), Paris, France; ^4^CENIR Platform, Institut du Cerveau (ICM), Paris, France; ^5^Department of Neurosurgery, APHP, Hôpitaux Universitaires Pitié-Salpêtriére/Charles Foix, Paris, France; ^6^Department of Neurosurgery, Brigham and Women's Hospital, Harvard Medical School, Boston, MA, United States

**Keywords:** somatotopy, diffusion MRI, tractography, stereotactic surgery, neuroanatomy

## Abstract

**Background:**

The subthalamic nucleus (STN) is an effective neurosurgical target to improve motor symptoms in Parkinson's Disease (PD) patients. MR-guided Focused Ultrasound (MRgFUS) subthalamotomy is being explored as a therapeutic alternative to Deep Brain Stimulation (DBS) of the STN. The hyperdirect pathway provides a direct connection between the cortex and the STN and is likely to play a key role in the therapeutic effects of MRgFUS intervention in PD patients.

**Objective:**

This study aims to investigate the topography and somatotopy of hyperdirect pathway projections from the primary motor cortex (M1).

**Methods:**

We used advanced multi-fiber tractography and high-resolution diffusion MRI data acquired on five subjects of the Human Connectome Project (HCP) to reconstruct hyperdirect pathway projections from M1. Two neuroanatomy experts reviewed the anatomical accuracy of the tracts. We extracted the fascicles arising from the trunk, arm, hand, face and tongue area from the reconstructed pathways. We assessed the variability among subjects based on the fractional anisotropy (FA) and mean diffusivity (MD) of the fibers. We evaluated the spatial arrangement of the different fascicles using the Dice Similarity Coefficient (DSC) of spatial overlap and the centroids of the bundles.

**Results:**

We successfully reconstructed hyperdirect pathway projections from M1 in all five subjects. The tracts were in agreement with the expected anatomy. We identified hyperdirect pathway fascicles projecting from the trunk, arm, hand, face and tongue area in all subjects. Tract-derived measurements showed low variability among subjects, and similar distributions of FA and MD values among the fascicles projecting from different M1 areas. We found an anterolateral somatotopic arrangement of the fascicles in the corona radiata, and an average overlap of 0.63 in the internal capsule and 0.65 in the zona incerta.

**Conclusion:**

Multi-fiber tractography combined with high-resolution diffusion MRI data enables the identification of the somatotopic organization of the hyperdirect pathway. Our preliminary results suggest that the subdivisions of the hyperdirect pathway projecting from the trunk, arm, hand, face, and tongue motor area are intermixed at the level of the zona incerta and posterior limb of the internal capsule, with a predominantly overlapping topographical organization in both regions. Subject-specific knowledge of the hyperdirect pathway somatotopy could help optimize target definition in MRgFUS intervention.

## Introduction

The hyperdirect cortico-subthalamic pathway is a set of white matter fibers sending direct inputs from the cortex to the subthalamic nucleus (STN) (1, 2). Hyperdirect pathway projections are sought to play a key role in the clinical outcomes of Deep Brain Stimulation (DBS) of the STN in Parkinson's disease (PD) patients (3–5). Several studies have demonstrated that direct stimulation of hyperdirect pathway fibers is involved in DBS therapeutic effects (4, 6–10). In particular, hyperdirect pathway projections from motor and premotor cortical areas are sought to be a major neural substrate modulated by STN DBS in addition to the STN itself (11). Recently, Magnetic Resonance guided Focused Ultrasound (MRgFUS) subthalamotomy has shown potential for improving motor symptoms and dyskinesias in PD patients (12–14). In that context, patient-specific knowledge of the topography of hyperdirect pathway fibers could help optimize target definition during planning of DBS and MRgFUS intervention. The anatomy of the hyperdirect pathway has been investigated in non-human primates (2, 15, 16). Animal experiments using anterograde tracers in monkeys have shown that the STN receives somatotopically organized projections from the primary motor cortex, and that these projections are arranged from medial to lateral in the order of hindlimb, forelimb and orofacial part (2, 3, 16). In rats, retrograde and anterograde tracing studies indicate that a subset of projections from the motor cortex innervate the STN and the striatum (17–19). In addition, anterograde tracing studies have shown that projections from the motor cortex are topographically organized with the rostral part of the lateral motor cortex projecting to the lateral portion of the rostral two-thirds of STN and the caudal part projecting to the ventral aspect of the middle third of STN (20).

Still, such neural tract-tracing techniques cannot be used to study brain connectivity on human subjects. User-defined holographic reconstructions of hyperdirect pathway fibers from structural MRI scans and histological data have been proposed to provide novel anatomical priors for human connectomic analysis (21). However, the reconstructed hyperdirect pathway fibers were defined based on scientific studies generated in the macaque brain, thus the approach presents the same limitations as other studies mapping results of non-human primates into human subjects (22). Diffusion MRI tractography enables the non-invasive exploration of white matter fibers at the individual patient scale. Recent advances in neuroimaging techniques have enabled identification of the trajectory of the hyperdirect pathway *in vivo* in individual subjects using single tensor deterministic tractography (10, 23–28), single tensor probabilistic tractography (29), multi-fiber probabilistic tractography (8, 10, 26, 27, 30–37) and generalized q-sampling imaging (38). Still, the internal organization of the hyperdirect pathway in the human brain remains unexplored. In this study, we seek to reconstruct hyperdirect pathways fibers projecting from the primary motor cortex using multi-fiber deterministic tractography and to investigate the internal organization of hyperdirect fascicles projecting from the trunk, arm, hand, face, and tongue area. We used MRI datasets from the Human Connectome Project as they offer the highest quality diffusion MRI data currently available to investigate brain connectivity. To the best of our knowledge, this is the first study of the somatotopy of the hyperdirect pathway in the human brain.

## Materials and Methods

### MRI Data Acquisition

We used high-resolution structural and diffusion MRI data from five healthy subjects (100307, 100408, 101915, 103414, 106016) of the Washington University, University of Minnesota, and Oxford University Human Connectome Project (WU- Minn HCP) consortium (39). The WU-Minn HCP scans were acquired on young healthy subjects (age 21–35) and represent the best neuroimaging data available for investigating the topography of the white matter in the human brain. The subjects were scanned on a customized Siemens 3.0 Tesla Skyra scanner using a 32-channel head coil and a customized gradient. The structural MRI data included T1-weighted and T2-weighted volumes acquired with the following parameters: T1-weighted: TE = 2.14 ms, TR = 2,400 ms, voxel size = 0.7 mm; T2-weighted: TE = 565 ms, TR = 3,200 ms, voxel size = 0.7 mm. The diffusion-weighted images were acquired using a single-shot 2D spin-echo multiband Echo Planar Imaging sequence with 90 gradient directions, 3 b-values (b1 = 1,000 s/mm^2^, b2 = 2,000 s/mm^2^, b3 = 3,000 s/mm^2^), 1.25 mm slice thickness and 1.25 mm image resolution (40, 41). The diffusion-weighted images used in this study had been processed for intensity normalization, eddy-current, patient-motion and EPI distortion correction and co-registered to the anatomical scans (42–44).

### MRI Data Analysis Workflow

Our data analysis workflow consisted of three steps: first, the segmentation of anatomical regions of interest, second the tractography reconstruction of hyperdirect pathway fibers, and third the analysis of the somatotopic organization of the hyperdirect pathway.

#### Segmentation of the Regions of Interest

We defined three sets of anatomical regions of interest (ROIs) in the primary motor cortex (M1), internal capsule (IC) and subthalamic nucleus (STN) using the 3D Slicer open-source platform for medical research (45). In the first set of ROIs, we outlined the precentral gyrus in a volume-rendered image of the T1-weighted scan. In the second set of ROIs, we generated a fractional anisotropy (FA) map and a diffusion-encoded color (DEC) map from the diffusion-weighted images using the SlicerDMRI extension of 3D Slicer (46, 47). We manually segmented the posterior limb of the IC in axial cross-sections of the FA map overlaid on the DEC map using the Segment Editor module of 3D Slicer. In the third set of ROIs, as the contours of the STN were not directly visible in the T1-weighted images, we used the automated atlas-based segmentation approach of the pyDBS software implemented in 3D Slicer (48). The method uses a 3D histological and deformable atlas of the basal ganglia that comprises 3D meshes of 80 structures identified on histological stainings from a post-mortem specimen (49). We deformed the Yeb atlas using a global-to-local registration approach to generate 3D meshes of the STN from the T1-weighted images (50). The meshes were subsequently voxelized in 3D Slicer to create isotropic ROIs with 0.3 mm voxel size.

#### Tractography Reconstruction

We applied a combination of diffusion MRI analysis tools that arose from the specific experience of our team. We used a multi-fiber ball-and-stick modeling to estimate the orientation of white matter fibers at each voxel from the diffusion-weighted MRI data. The ball-and-stick model is a multi-compartment approach constrained to include an isotropic “ball” compartment and multiple anisotropic “sticks” compartments (51). The model was fitted to the diffusion-weighted MRI data using a Bayesian estimation procedure to robustly estimate fiber orientations and volume fractions, as well as their total count (52). Parameter settings included the continuous exponential approach for multi-shell data and a maximum of three fiber compartments per voxel.

To reconstruct the hyperdirect pathway fibers using the ROIs described above, we used a multi-fiber streamline tractography algorithm with a model-based interpolation framework (53, 54) from the Quantitative Imaging Toolkit (QIT) (55). The ball-and-stick models were interpolated during bundle tracking using a data-adaptative kernel regression framework with a spatial bandwidth of 1.0 mm, model selection parameter λ = 0.9999, and up to three fiber compartments (54). Fiber tracking was performed using a step size of 0.5 mm, and 25 seeds per voxel in a one-voxel neighborhood surrounding the M1 and STN ROIs. Hyperdirect pathway fibers were retained only if they intersected the M1, IC and STN ROIs. Tracts were terminated upon reaching the STN or M1 ROIs, when the angle changed more than 55 degrees, or when a compartment's volume fraction dropped below 0.05. We stopped the fibers at the STN surface as the image resolution was not sufficient to follow the tracts inside the nucleus. Thus, by design this aspect of the hyperdirect pathway termination is not covered in this study.

### Evaluation by Neuroanatomy Experts

Two expert neuroanatomists (J.Y. and C.F.) with over 30 years of experience performed qualitative evaluation of the anatomical accuracy of the hyperdirect pathway. For each subject, the tractography reconstructions were loaded in 3D Slicer along with the structural MRI scans, diffusion-weighted MRI scans, and 3D models of the subthalamic nucleus. The anatomical accuracy of the tracts was evaluated based on the similarity between the tractography reconstructions and known neuroanatomy using three criteria: the topographical localization of each tract; the start and end region of the fascicles, and the specific shape of the bundles. The experts assigned a score ranging from 5 (excellent) to 1 (poor) averaged on the criteria used for the review of each tract.

### Somatotopic Organization

To investigate the internal organization of the reconstructed pathways, we defined five primary motor cortex ROIs in a 3D volume-rendered image of the T1-weighted scan. The ROIs were placed in the trunk, arm, hand, face, and tongue motor homunculus in the precentral gyrus of each hemisphere using the Markups module of 3D Slicer. First, we identified the “hand-knob” sign in the precentral gyrus to place the hand motor ROI (56). Second, we positioned the arm ROI medial to the hand ROI. Third, the trunk ROI was placed medial to the arm ROI and close to the midline of the brain. Fourth, we placed the face ROI lateral to the hand ROI in the lower portion of the precentral gyrus in the section of the top of the lateral ventricles (57). Fifth, the tongue ROI was positioned in the most lateral portion of the precentral gyrus in the section just above the Sylvian fissure (57). We used the SlicerDMRI extension of 3D Slicer to extract the fascicles arising from the trunk, arm, hand, face, and tongue ROIs from the reconstructed hyperdirect pathway.

### Tract-Derived Measurements

To assess the variability among subjects, we calculated the fractional anisotropy (FA) and mean diffusivity (MD) of the envelope of the trunk, arm, hand, face and tongue hyperdirect pathway fascicles. We computed the envelope of the fascicles by converting the streamlines into voxel wise binary label maps with label = 1 when a tract was detected in a voxel and label = 0 when no tract was detected. The envelope, FA volume, and MD volume for each fascicle were calculated using the SlicerDMRI extension of 3D Slicer. In addition, to investigate the segregation of hyperdirect pathway fascicles, we segmented the contour of each fascicle in two axial slices at the level of the internal capsule and zona incerta superior to the subthalamic nucleus, and we computed the centroid of each contour.

### Quantitative Analysis

We performed a statistical analysis of the scores given by the neuroanatomy experts and the tract-derived measurements. We evaluated the degree of agreement between the experts using the intraclass correlation coefficient (ICC) and we computed the internal consistency of the scores using Cronbach's alpha reliability analysis (58). To evaluate the variability among subjects, we computed the average, maximum and standard deviation of FA and MD values for the fascicles associated with the trunk, arm, hand, face, and tongue M1 areas. Finally, to investigate the segregation of hyperdirect pathway fibers, we computed the Dice Similarity Coefficient (DSC) of overlap between the contours of the trunk, arm, hand, face, and tongue fascicles segmented in the axial slices at the level of the internal capsule and zona incerta. We compared the relative anterior-posterior orientation of the centroids of the contours to assess the spatial arrangement of the fascicles.

## Results

**Figure 1:**
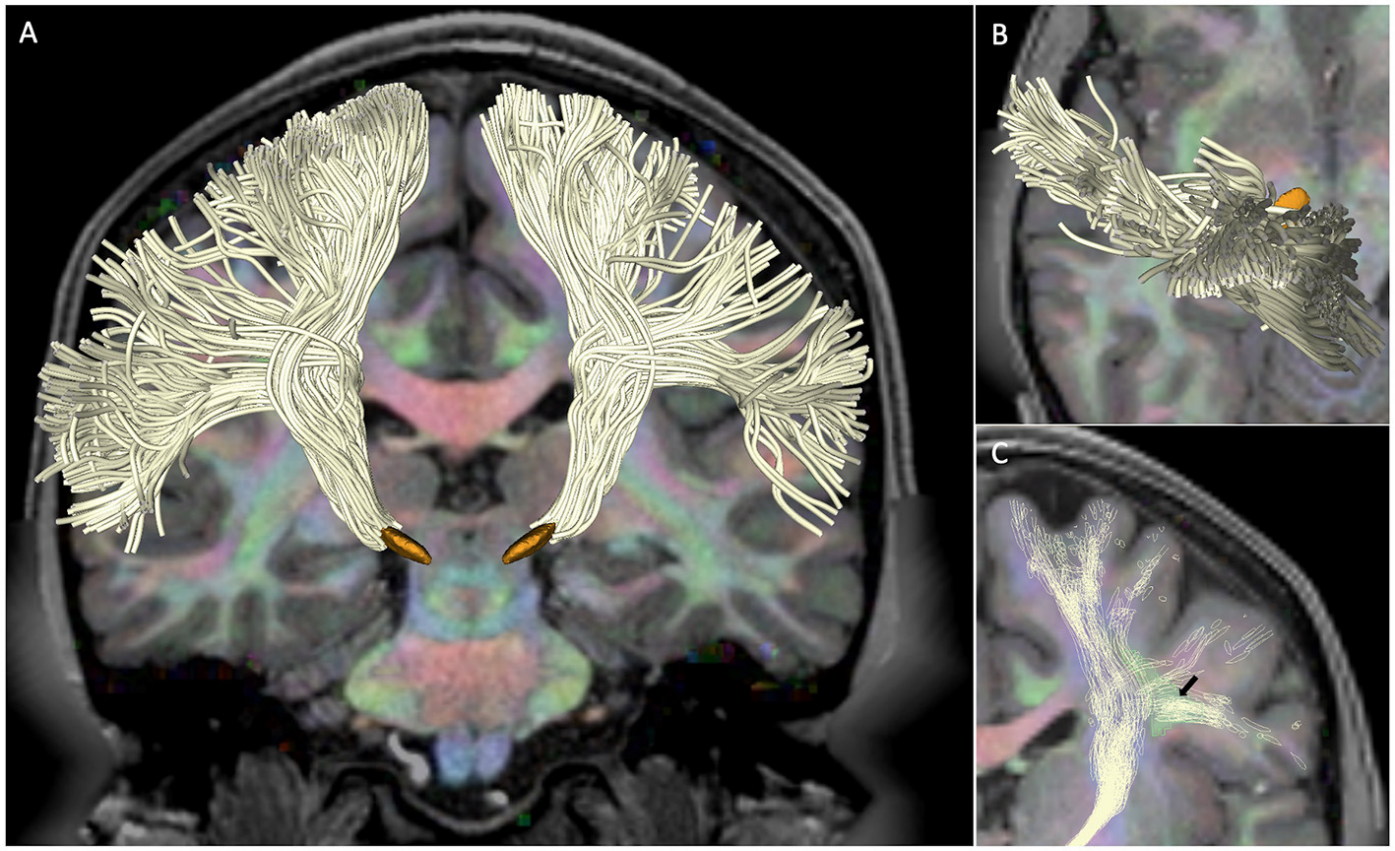
Hyperdirect pathway in subject 100307. The figure shows the tractography reconstruction of hyperdirect pathway fibers projecting from the primary motor cortex to the subthalamic nucleus. The tracts (white) are displayed on a diffusion encoded color map overlaid on a T1-weighted image with 3D models of the subthalamic nucleus (orange) for anatomical reference. **(A)** Anterior 3D view of the hyperdirect pathway. **(B)** Superior 3D view of hyperdirect pathway fibers descending from the primary motor cortex. **(C)** Anterior 3D view of the crossings of hyperdirect pathway fibers with the superior longitudinal fasciculus. The arrow points at the intersection of lateral projections of the hyperdirect pathway with a cross-section of the superior longitudinal fasciculus (green).

We successfully reconstructed hyperdirect cortico-subthalamic fibers connecting the primary motor cortex to the ipsilateral STN in all five subjects. The hyperdirect pathway fibers presented a fan shape configuration with fibers arising from the whole extend of the precentral gyrus, converging into the corona radiata, descending through the posterior limb of the internal capsule, and terminating in a compact stem entering the subthalamic nucleus. Figure 1 shows the 3D tractography reconstruction in a single subject. The use of an advanced fiber models enables the partial identification of complex fibers crossings of the hyperdirect pathway with the superior longitudinal fasciculus (Figure 1C).

The evaluation of the hyperdirect pathway fibers by two neuroanatomical experts demonstrated that the tractography reconstructions were in agreement with the expected anatomy with an average score of 3.7 ± 0.92. The ICC score was 0.44 and the Cronbach's alpha score was 0.91 with 95% confidence interval of [0.67, 0.98] which showed a good level of agreement between the experts.

**Figure 2:**
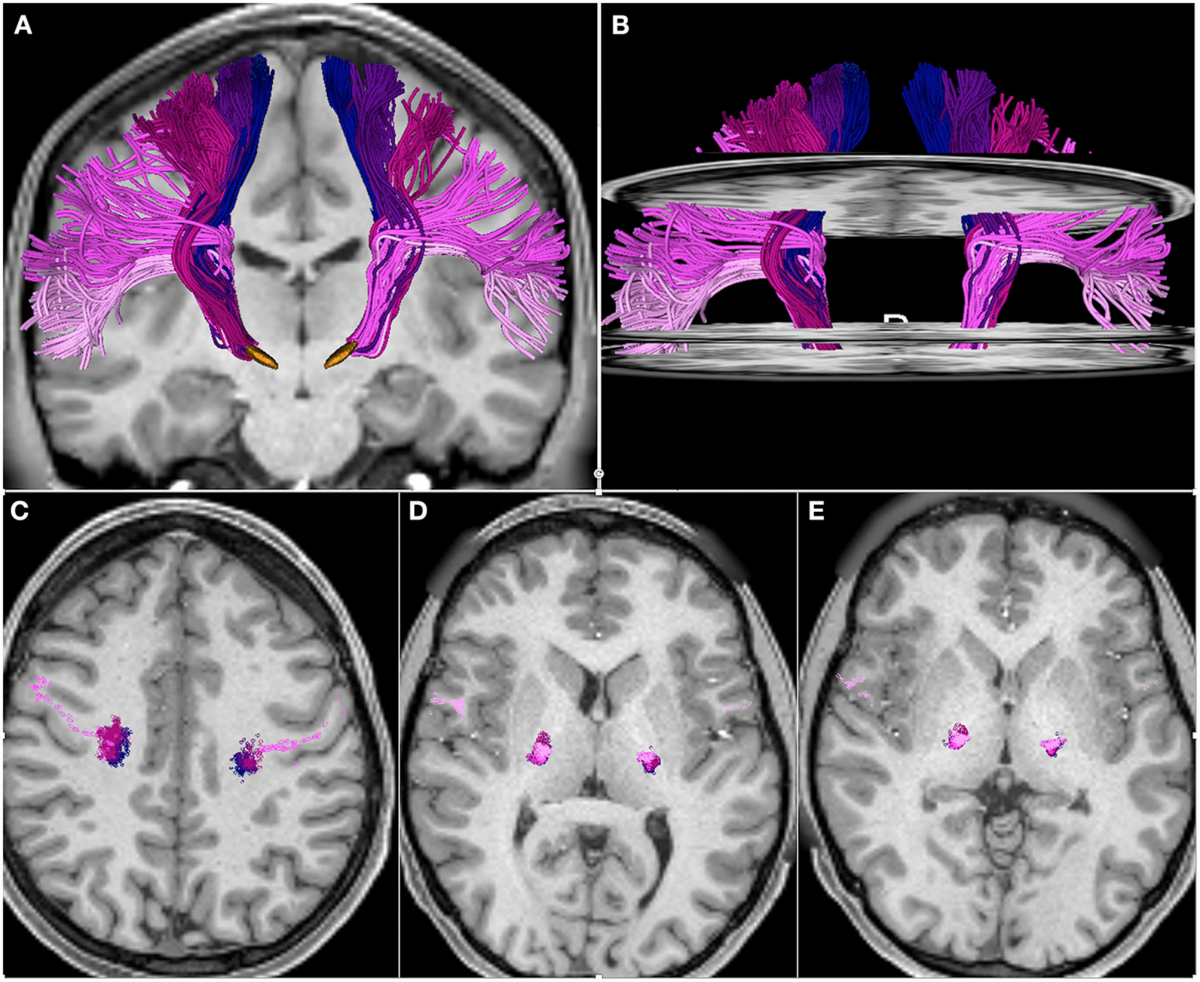
Somatotopic organization of hyperdirect pathway fibers projecting from the primary motor cortex trunk, arm, hand, face, and tongue area. The tracts are displayed on axial and coronal T1-weighted images. **(A)** 3D anterior view of hyperdirect pathway fibers with 3D models of the subthalamic nucleus (orange). **(B)** 3D anterior view of hyperdirect pathway fibers with axial T1-weighted images at the level of the coronal radiata, internal capsule and zona incerta. **(C–E)** Intersection of hyperdirect pathway fibers with an axial T1-weighted image at the level of the corona radiata **(C)**, posterior limb of the internal capsule **(D)**, and zona incerta **(E)**. The three axial slices in **(B)** correspond to the axial images displayed in **(C–E)**. The fibers are colored according to the motor regions: trunk (blue), arm (dark purple), hand (dark pink), face (light purple), tongue (light pink).

**Figure 3:**
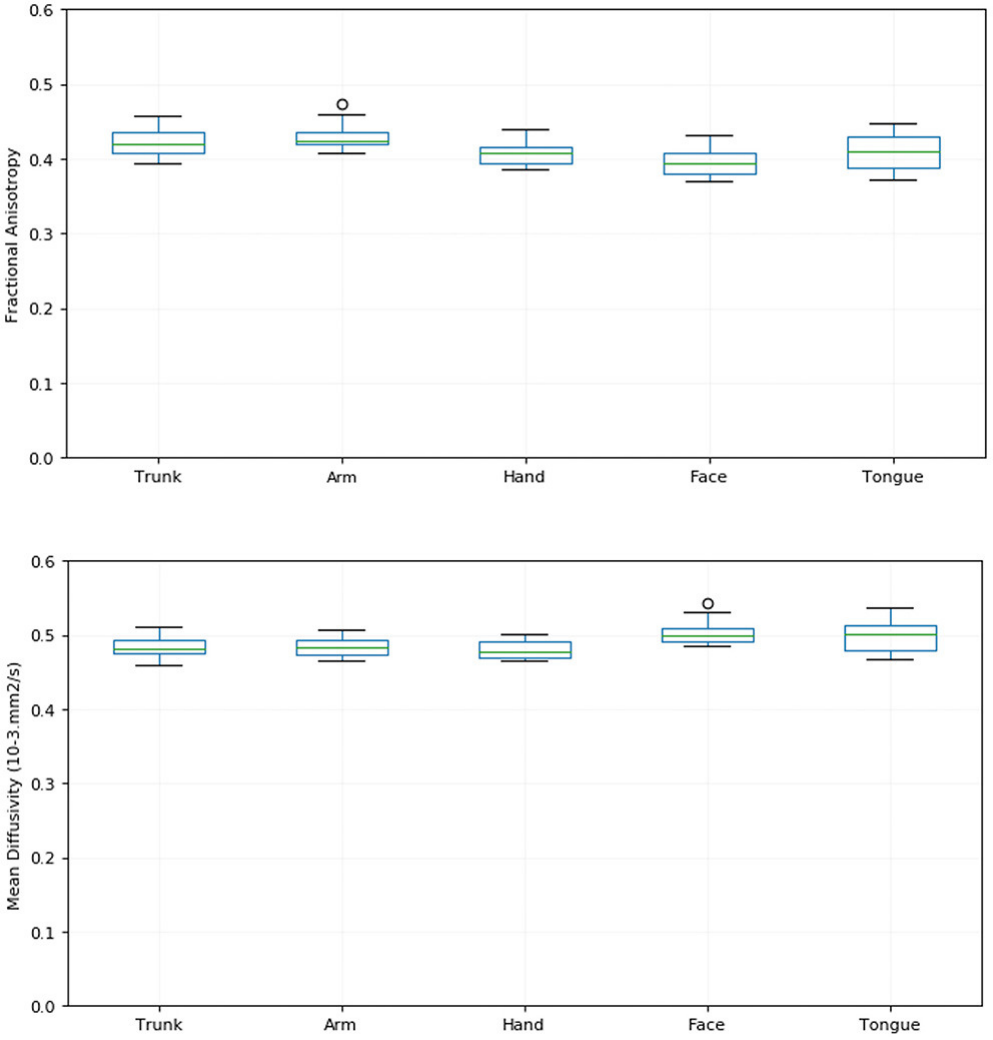
Tract-derived measurements across subjects. The figure shows box plots of the fractional anisotropy (FA) **(top)** and mean diffusivity (MD) **(bottom)** of hyperdirect pathway fascicles projecting from the trunk, arm, hand, face, and tongue motor area for all subjects. The lines in each box correspond to the median (green), interquartile range (blue), minimum and maximum (black) values of the tract-derived measurements.

We identified fascicles projecting from trunk, arm, hand, face, and tongue M1 area in all subjects. Figure 2 shows the somatotopic organization of hyperdirect pathway fibers in a single subject. The spatial arrangement of hyperdirect pathway fascicles was anterolateral in the corona radiata and predominantly overlapping at the level of the internal capsule and zona incerta. Tract-derived measurements showed low variability among subjects, and similar distributions of FA and MD values among the tracts projecting from the different motor regions (Figure 3).

Table 1 summarizes the Dice Similarity Coefficient (DSC) of spatial of overlap values between the trunk, arm, hand, face and tongue fascicles in the internal capsule and zona incerta. Overall, the tracts in the internal capsule showed an average overlap of 0.63 with a maximum average overlap of 0.83 for the fascicles projecting from the arm and hand area, and a minimum average overlap of 0.47 for the fascicles projecting from the trunk and tongue area. In the zona incerta, the tracts showed an average overlap of 0.65 with a maximum average overlap of 0.81 for the fascicles projecting from the arm and hand area, and a minimum average overlap of 0.50 for the fascicles projecting from the hand and tongue area. The analysis of the position of the centroid of the tracts in the internal capsule showed that the most anterior position was occupied by tracts projecting from the tongue (30%) and face (30%) area, while the most posterior position was occupied by tracts projecting from trunk (70%) and tongue (20%) area. In the zona incerta, the most anterior position was occupied by tracts projecting from the hand (40%) and face (40%) area; the most posterior position was occupied by tracts projecting from the tongue (50%) and trunk (30%) area.

**Table 1:** Dice Similarity Coefficient of overlap of hyperdirect pathway fascicles in the internal capsule and zona incerta.

**DSC**		**Tr-A**	**Tr-H**	**Tr-F**	**Tr-To**	**A-H**	**A-F**	**A-To**	**H-F**	**H-To**	**F-To**
IC	Mean	0.8	0.74	0.63	0.47	0.83	0.7	0.49	0.68	0.48	0.49
	Std	0.07	0.03	0.09	0.21	0.08	0.13	0.18	0.14	0.12	0.18
ZI	Mean	0.76	0.71	0.67	0.54	0.81	0.73	0.53	0.68	0.50	0.57
	Std	0.09	0.09	0.13	0.21	0.09	0.14	0.20	0.10	0.21	0.20

*The table shows the mean and standard deviation (std) of the Dice Similarity Coefficient (DSC) of spatial overlap of the fascicles projecting from the trunk (Tr), arm (A), hand (H), face (F), and tongue (To) motor area displayed in Figure 2. Results show that the fascicles predominantly overlap with a minimum DSC average of 0.47 in the internal capsule (IC) and 0.50 in the zona incerta (ZI)*.

## Discussion

Our exploratory study investigated the somatotopic organization of hyperdirect pathway projections from the primary motor cortex to the subthalamic nucleus (STN) on healthy subjects of the Human Connectome Project. We have shown that multi-fiber tractography and high-resolution multi-shell diffusion-weighted MRI data enable the identification of white matter fascicles connecting the trunk, arm, face, hand, and tongue primary motor cortex area to the subthalamic nucleus. We demonstrated that the use of an advanced fiber model enables the identification of lateral projections of the hyperdirect pathway from the face and tongue motor area, as well as some complex fibers crossings with the superior longitudinal fasciculus. The evaluation of the anatomical accuracy by neuroanatomy experts demonstrated that the topography of the reconstructed tracts was in agreement with known neuroanatomy. The analysis of tract-derived measurements demonstrated a low level of variability among subjects. We found an anterolateral somatotopic arrangement in the most superior section of the hyperdirect pathway. In the internal capsule, hyperdirect pathway fibers projecting from different motor areas showed an average overlap of 0.63, with the most anterior position occupied by tongue and face fibers and the most posterior position occupied by trunk and tongue tracts. In the zona incerta, the average overlap was 0.65 with the most anterior position occupied by hand and face fibers and the most posterior position occupied by tongue and trunk fibers. Our preliminary tractography results suggest that the subdivisions of the hyperdirect pathway are intermingled in the zona incerta and posterior limb of the internal capsule, with a predominantly overlapping topographical organization in both regions. Tract-tracing experiments in monkeys have previously reported anatomical overlap of projections from the frontal cortex in subcortical structures: Selemon et al. observed topographical overlap and interdigitation of corticostriatal projections in the ventromedial striatum (59); in the pedunculopontine nucleus, Matsumara et al. have shown that the somatotopic representations of projections from motor-related areas are intermingled rather than segregated (60).

Our tractography findings in the hyperdirect pathway are consistent with observations from two previous experimental studies on the structure and function of the STN in the human brain. In 2002, an anatomical study using calbindin labeling demonstrated that the subdivisions of the STN are separated by functional gradients, not by sharp boundaries (61). In 2007, a DBS study on PD patients suggested that the STN serves as a nexus for the integration of motor, cognitive and emotional components of behavior and that these functional modalities are not processed in a segregated manner (62). These two studies on the lack of anatomical and functional segregation of components of the STN are in agreement with our observations on the overlap of hyperdirect pathway fibers projecting from different cortical regions. Furthermore, a cytoarchitectural study of STN neurons in the human brain has shown that the dendrites of STN neurons extend up to 1,200 μm to neighboring territories, which suggests a convergence of inputs from different cortical areas on individual neurons of the nucleus (63). This convergence has been used in the model of focused selection and inhibition of competing motor programs by the basal ganglia (64). In this model, when voluntary movement is generated, the motor cortex uses the hyperdirect pathway to send a short-latency signal to the whole STN, which causes a fast and widespread excitation of the globus pallidus pars interna (GPi) and substantia nigra pars reticulata (SNpr) resulting in an inhibition of competing motor mechanisms that would interfere with the desired movement (64). Simultaneously, the motor cortex creates a focused excitation of the striatum through the direct pathway, which causes a focused inhibition of specific GPi and SNpr neurons, followed by a focused excitation of neurons in the thalamus and cortex allowing the desired movement to proceed (64). The convergence of inputs from different cortical areas suggests that the STN might not be topographically organized to preserve the somatotopy of the motor cortex (62), which is in agreement with our findings on the topography of corticosubthalamic fibers.

To the best of our knowledge, this is the first study of the somatotopy of the hyperdirect pathway in the human brain. Tractography studies have investigated the somatotopy of the pyramidal pathway using single-tensor deterministic tractography (65–69), single-tensor probabilistic tractography (70–73), and two-tensor probabilistic tractography (74). These studies have reported a segregation of corticospinal tract fibers (65–74) and an overlap of corticobulbar tract fibers (74). While the pyramidal tract is adjacent to the hyperdirect pathway, the two white matter bundles are anatomically different. Pyramidal projections from the primary motor cortex are composed of corticospinal projections to the spine and corticobulbar projections to motor nuclei of cranial nerves, whereas hyperdirect pathway projections from the primary motor cortex terminate in the subthalamic nucleus. In addition, the hyperdirect pathway and the pyramidal pathway have opposite roles in DBS outcomes: direct stimulation of the hyperdirect pathway is involved in DBS therapeutic effects (4, 6–10), whereas the spread of current to the pyramidal tract can trigger pyramidal tract side effect (75). These anatomical and functional differences are a potential explanation of the differences between the somatotopy we observed in our study and the somatotopies reported in pyramidal tract studies.

The somatotopic organization of the STN has been described in healthy monkeys using invasive neural tracing techniques. These studies have shown that the primary motor cortex projects to the whole extend of the STN (1, 16), and neural tracers have revealed a somatotopic arrangement with lower-limb cells located medially to upper-limb cells, and the face area located in the most lateral zone of the nucleus (1, 76, 77). Several groups have investigated the somatotopic organization of the STN in Parkinson's disease patients using microelectrode recordings (MERs) to identify movement related cells (MRCs) during DBS surgery (78–82). In these studies, most MRCs were detected in the dorsolateral portion of the STN. Rodriguez-Oroz et al. identified a somatotopic distribution similar to the distribution in healthy monkeys, with cells associated in the lower limb located in the upper dorsal third and centromedian portion, and cells associated with the upper limb located in the dorsal two-thirds and lateral region of the STN (78). Abosch et al. found movement-related neurons located throughout the STN, including the ventral portion of the nucleus despite a rostrodorsal clustering of the cells, and reported an absence of clear somatotopic relationship of limb representation (79). Theodosopoulos et al. reported arm-related cells located laterally and at the rostral and caudal poles of the STN, and leg-related cells located medially and centrally (80). Romanelli et al. found that lower extremity–related cells were located medial and ventral to upper extremity–related cells (81). Sasaki et al. showed that cells responding to the upper limbs were more commonly observed in the lateral, anterior, and superior regions of the STN, and that cells associated with the distal joints were located above those associated with the proximal joints, in both upper and lower limbs (82). While somatotopic findings in Parkinson's disease patients may not be generalized to healthy subjects, these electrophysiology results in the STN contrast with our tractography findings. Although we did not investigate the somatotopy of the fibers inside the STN due to the presence of multiple fiber crossings that pose technical limitations to tractography algorithms, our results in the zona incerta immediately superior to the STN show a predominant overlap of hyperdirect fascicles projecting from different motor regions without any clear segregation of the fibers. These discrepancies between MERs findings and tractography results might arise from the difference in scale between the two techniques: the 10 μm diameter of the tip of a microelectrode enables single neuronal response recordings whereas the 1.25 mm voxel size of a diffusion-weighted MRI dataset contains over 100,000 axons. In addition, hyperdirect pathway fibers projecting from large regions of the primary motor cortex homunculus are compacted into a relatively small space at the level of the internal capsule and zona incerta. The use of smaller voxel size may help further establish whether homunculus organization observed in the primary motor cortex is maintained as hyperdirect pathway fibers descend to subcortical areas, as suggested by electrophysiology studies of the STN, or whether intermingling of fibers occurs.

Our study presents several limitations. First, we used a small number of subjects as our goal was to explore the feasibility of identifying the somatotopic organization of the hyperdirect pathway using multi-fiber tractography and the highest quality neuroimaging data available. We showed that white matter bundles projecting from the trunk, arm, hand, face and tongue motor areas could be consistently identified in all subjects. Future work using a larger cohort will enable us to investigate the anatomical variability of hyperdirect pathway projections from the primary motor cortex. Second, we used voxelized tracts to evaluate the variability of tract-derived measurements across subjects. Converting streamlines into a voxel grid can introduce small inaccuracies due to partial volume effects. Third, while we used an advanced multi-fiber model and high-resolution diffusion-weighed data, our tractography results include false-negative tracts in the hand and face fascicles as seen in Figure 2A. These false-negative tracts are likely due to complex fiber crossings with the dorsal superior longitudinal fasciculus which interconnects the frontal and parietal lobe (83). While our multi-fiber model enabled the resolution of some of the crossings of hyperdirect fibers with the superior longitudinal fasciculus, the large extent of the pathway still poses technical challenges to fiber tracking. We conducted our analysis using HCP diffusion MRI data which are the best neuroimaging data currently available to study the connectivity of the human brain. The 1.25 mm-voxels of the diffusion-weighted images nevertheless contain a large number of axons belonging to different white matter bundles. Future work with a higher number of compartments will enable us to refine the tractography reconstruction of complex fiber crossings of the hyperdirect pathway with the superior longitudinal fasciculus. Further developments also include the exploration of other advanced tractography methods such as multi-fiber unscented Kalman Filter tractography (47). Finally, a comparison of histological data with our tractography findings would help investigate the topography of hyperdirect pathway fibers in the internal capsule and in the zona incerta.

Diffusion MRI tractography provides a non-invasive window on the architecture of the human brain white matter. While tractography reconstructions enable 3D visualization of the location and trajectory of white matter pathways, the technique still presents limitations for neurosurgical decision-making (84). As mathematical models of diffusion and fiber tracking algorithms are continuously refined by the medical image computing research community, diffusion MRI tractography has the potential to become part of the apparatus of brain mapping tools to help understand the clinical effects of electrical stimulation and focused ultrasound lesioning in motor disorders patients. Tractography reconstructions of the pyramidal tract, medial lemniscus and dentatorubrothalamic tract have provided innovative tractography-based approaches for targeting the ventral intermediate nucleus during MRgFUS intervention in Essential Tremor patients (85–89). Recent studies using tractography reconstructions of the hyperdirect pathway on motor disorder patients have revealed the potential of the technique to assist with Deep Brain Stimulation intervention in Parkinson's Disease patients (8, 25–28, 34, 35, 37, 38). The HCP diffusion MRI scans represent the state-of-the-art data for characterizing structural human brain connectivity. As transcranial focused ultrasound subthalamotomy is being investigated for unilateral treatment of motor symptoms and dyskinesias in Parkinson's Disease patients (12–14), our study aimed at investigating tractography reconstructions of the hyperdirect pathway that could become available to assist with target definition during MRgFUS intervention in the near future.

## Conclusion

Our study shows that advanced multi-fiber tractography techniques combined with high-resolution diffusion MRI data enable 3D reconstruction of the whole extent of hyperdirect projections from the primary motor cortex to the STN and the identification of cortico-subthalamic fascicles arising from the trunk, arm, hand, face, and tongue area. Our preliminary tractography results suggest that the subdivisions of the hyperdirect pathway are intermingled in the zona incerta and posterior limb of the internal capsule, with a predominantly overlapping topographical organization in both regions. Diffusion MRI tractography is a clinical research tool that holds promise for identifying the location and trajectory of white matter pathways during stereotactic surgery. Knowledge of the somatotopic organization of the hyperdirect pathway at the individual patient scale could provide clinically relevant information for planning stereotactic surgery of the STN, and contribute to advancing the understanding of the therapeutic mechanisms of action of MRgFUS and DBS. White matter maps of the hyperdirect pathway in healthy subjects could help evaluate potential alterations of the somatotopy of different body parts in Parkinson's disease patients, and thus expand our understanding of the pathophysiology of the disease.

## Data Availability Statement

The raw data supporting the conclusions of this article will be made available by the authors, without undue reservation.

## Ethics Statement

The study used publicly available data from the Human Connectome Project. Ethical review and approval was not required for the study on human participants in accordance with the local legislation and institutional requirements. Written informed consent for participation was not required for this study in accordance with the national legislation and the institutional requirements.

## Author Contributions

SP, RC, JY, CF, EB, GC, and RK: substantial contributions to the conception or design of the work. SP, RC, SFV, EB, and RK: substantial contributions to the data analysis. SP, JY, CF, GC, CK, and RK: substantial contributions to the interpretation of data with anatomical expertise. SP, RC, GC, and RK: substantial contributions to drafting the work. SP, RC, JY, EB, CF, CK, GC, and RK: substantial contributions to revising the work. All authors agreement to be accountable for all aspects of the work in ensuring that questions related to the accuracy or integrity of any part of the work are appropriately investigated and resolved.

## Funding

This work was partially funded by the Neuroimage Analysis Center (NIH P41EB015902 to SP and RK) and the National Center for Image-Guided Therapy (NIH P41EB015898 to RK). SP was supported in part by grant number 2021-237549(5022) from the Chan Zuckerberg Initiative DAF, an advised fund of Silicon Valley Community Foundation. SP and RK were supported in part by the Lymph Node Quantification System for Multisite Clinical Trials (NIH R01CA235589). RC was supported in part by grant number 2020-225670 from the Chan Zuckerberg Initiative DAF, an advised fund of Silicon Valley Community Foundation.

## Conflict of Interest

The authors declare that the research was conducted in the absence of any commercial or financial relationships that could be construed as a potential conflict of interest. The handling editor declared a past collaboration with one of the authors GC.

## Publisher's Note

All claims expressed in this article are solely those of the authors and do not necessarily represent those of their affiliated organizations, or those of the publisher, the editors and the reviewers. Any product that may be evaluated in this article, or claim that may be made by its manufacturer, is not guaranteed or endorsed by the publisher.
